# Diversity of Major Yield Traits and Nutritional Components Among Greenhouse Grown Chickpea (*Cicer arietinum* L.) Breeding Lines, Landraces, and Cultivars of Different Origins

**DOI:** 10.3390/plants13213078

**Published:** 2024-11-01

**Authors:** Yu-Mi Choi, Hyemyeong Yoon, Myoung-Jae Shin, Sukyeung Lee, Jungyoon Yi, Xiaohan Wang, Kebede Taye Desta

**Affiliations:** 1 National Agrobiodiversity Center, National Institute of Agricultural Sciences, Rural Development Administration, Jeonju 54874, Republic of Korea; cym0421@korea.kr (Y.-M.C.); mmihm@korea.kr (H.Y.); smj1204@korea.kr (M.-J.S.); wangxiaohan0530@aliyun.com (X.W.); 2 International Technology Cooperation Center, Technology Cooperation Bureau, Rural Development Administration, Jeonju 54875, Republic of Korea; reset00@korea.kr

**Keywords:** chickpea, diversity, genetic resource, genotype, multivariate analysis, nutrition, yield

## Abstract

This study analyzed the diversity of major yield traits and nutritional components across 122 chickpea breeding lines, cultivars, and landraces of different origins. All parameters showed significant variations, with a variance ranging from 4.61% in days to maturity (DM) to 43.04% in oleic acid. Six accessions, including CP021, CP022, CP026, CP037, CP066, and CP109, outperformed in yield traits and nutritional value. Origin significantly affected all phenotypic traits except total fatty acid contents, with Indian and Ukrainian accessions demonstrating contrasting performances. Most traits, except for the number of seeds per pod (SPP), palmitic acid, and total fatty acid contents, differed significantly among breeding lines, cultivars, and landraces. Breeding lines were the earliest to flower and to mature with average days to flowering (DF) of 50.23 days and DM of 101.50 days. They also had the highest average SPP, number of pods per plant (PPP), total seeds per plant (TSPP), total protein, crude fiber, dietary fiber, linoleic acid, and linolenic acid contents making them preferable for high yield and nutrition. Hierarchical cluster analysis classified the chickpea accessions into seven clusters, showing significant variations in yield traits and nutritional components. Principal component and Pearson’s correlation analyses indicated positive correlations between DM and DF, and between SPP, PPP, and TSPP. Nutritional components also displayed varying associations, with a notable negative correlation between oleic and linoleic acids, the two essential fatty acids. Overall, this study showed the diversity of key phenotypic traits in chickpea breeding lines, cultivars, and landraces of different origins. The significant effects of genotype and origin differences on these traits could be used as a basis for future metabolomics and genomics research.

## 1. Introduction

Chickpea (*Cicer arietinum* L.) is one of the most widely cultivated and economically important legumes [1]. According to the latest FAO data, the global chickpea production has increased from 10.84 M tones in 2010 to 18.10 M tones in 2022 signifying its role in global food security and agricultural sustainability [2]. Moreover, chickpea’s adaptability to various climatic regions and its resistance to unfavorable weather conditions could also have contributed to its increased production. These all make it a staple crop in several countries across Asia, Africa, Europe, Australia, and North and South America [3,4]. Currently, India is the leading chickpea producing country followed by Australia, Turkey, and Ethiopia [2]. In Korea, unlike other legumes such as soybean and mung bean, the production of chickpea is not widely practiced. However, there is an increasing interest in utilizing chickpea and chickpea products for daily consumption [5]. The latest national data showed that chickpea import has increased from 2485.0 tons in 2017 to 4776.9 tons in 2022 showing a two-fold increase within five years alone (http://unipass.customs.go.kr, accessed on 25 October 2024).

In addition to its agricultural importance, chickpea is known for its remarkable nutritional profile and health benefits [6]. Chickpea seeds are rich in protein, vitamins, fiber, and minerals, among others [7,8]. Furthermore, they are rich in bioactive compounds such as polyphenols and flavonoids, which have been known to exhibit several bioactivities such as anti-inflammatory, antioxidant, and anti-cancer properties, among others [9,10]. The abundance of fatty acids with different degrees of saturation also makes them a good source of plant-based healthy oil. These qualities make chickpea seeds a valuable addition to daily consumption, including in vegan diets [11,12].

The metabolite content and agronomic traits of crops, in general, are influenced by different factors associated with genetic differences, environmental conditions, and agricultural practices [13,14]. Several studies have shown the effects of biotic and abiotic stresses, soil quality, origin, cultivation years, and post-harvest practices on both yield traits and nutrient composition in different legumes including chickpea [13,15,16,17]. Such studies have led to the selection of superior varieties that have the potential to be utilized in food industries and breeding programs, as well as used for future conservation and dissemination to farmers [18,19,20]. Recent research trends in chickpea also focused on investigating the effects of different factors related to genotype and environment on their agronomic performances as well as nutritional qualities [20,21]. This area of study on field and/or greenhouse-cultivated genetic materials not only enhances our understanding of the diversity found in chickpea but also aids in selecting varieties that are better suited to specific growth conditions, used for conservation, and utilized in food industries [18,22].

With the growing demand for food resources and the challenge of climate change, studies that investigate the diversity found in chickpea genetic materials are crucial and regularly required as highlighted before [16,17,18,19,20]. Moreover, studying the influence of different factors on their yield traits and nutritional qualities is equally important. In this regard, studying a large collection of chickpea genotypes offers numerous benefits, particularly in providing a wide range of options to identify key traits associated with high yield and nutritional value [14]. Genebanks play a crucial role since they possess a large collection of genetic materials of diverse biological status and provide several options for selecting varieties with desirable qualities [23,24]. Our genebank at the National Agrobiodiversity Center (Jeonju, Republic of Korea) contains a global collection of about 330 diverse chickpea genetic materials of different origins thus far. These resources need to be comprehensively characterized and investigated before being utilized in breeding, disseminated to farmers and food industries, or conserved for future use [23,24]. Accordingly, this study aimed to assess the diversity of key phenotypic traits related to growth periods (days to flowering (DF), days from flowering to maturity (DFM), and days to maturity (DM)), yield (number of pods per plant (PPP), number of seeds per pod (SPP), number of seeds per plant (TSPP), and one hundred seeds weight (HSW)), nutrient levels (crude fiber, dietary fiber, total fat, total protein contents), and fatty acids (palmitic acid, stearic acid, oleic acid, linoleic acid, and linolenic acid) among 122 greenhouse grown chickpea accessions including 10 cultivars, 48 landraces, and 64 breeding lines collected from Bulgaria, Georgia, India, Myanmar, and Ukraine. Supported by multivariate statistical tools, the variations of each phenotypic trait among the different chickpea materials and country of origin were statistically investigated. To the best of our knowledge, this study is the first to statistically investigate the variations of yield traits and nutritional components among greenhouse-grown chickpea cultivars, landraces, and breeding lines of different origins. Overall, the results of this study may provide valuable insights into the diversity of yield traits and nutritional parameters in chickpea genetic resources and could initiate future metabolomics and genomic studies.

## 2. Results and Discussion

### 2.1. Variation of Growth Period and Yield Traits

**Figure 1:**
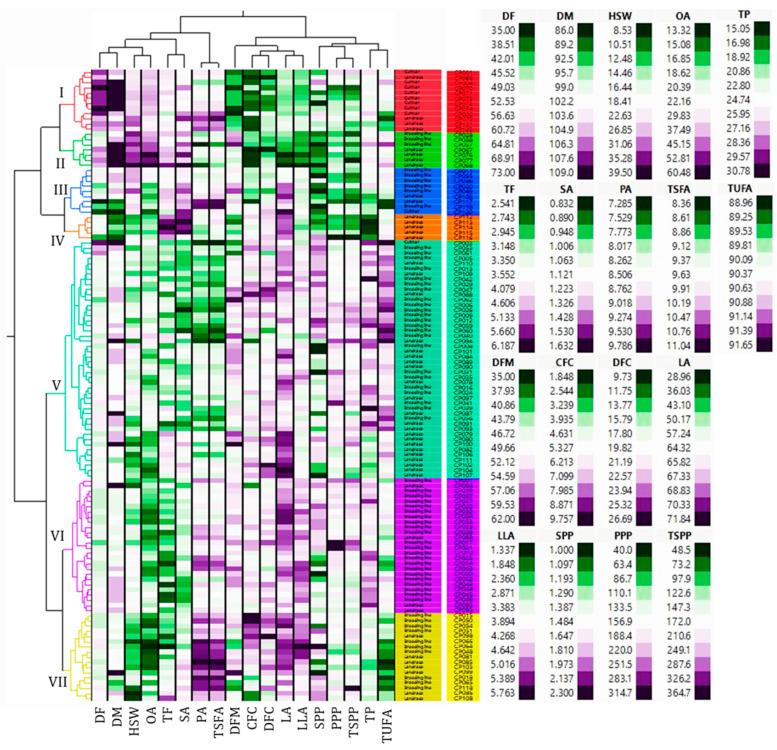
Heatmap showing two-way hierarchical cluster analysis of chickpea accessions. CFC: Crude fiber content, DFM: Days from flowering to maturity, DF: Days to flowering, DFC: Dietary fiber content, DM: Days to maturity, HSW: One-hundred seeds weight, LA: Linoleic acid, LLA: Linolenic acid, OA: Oleic acid, PA: Palmitic acid, PPP: Number of pods per plant; TSPP: Total seeds per plant; SA: Stearic acid, SPP; Number of seeds per pod, TF: Total fat, TP: Total protein, TSFA: Total saturated fatty acid, TUFA: Total unsaturated fatty acid.

The variations of the yield traits along with growth periods across the 122 chickpea accessions are presented in the HCA plot (Figure 1) while the statistical data is summarized in Table 1.

The coefficient of variation (CV) ranged from 4.61% in DM to 34.91% in TSPP showing significant variation among the chickpea genetic materials (*p* < 0.05). DF, DFM, and DM were in the ranges of 35.00–73.00, 35.00–62.00, and 86.00–109.00 days, respectively. Likewise, SPP ranged between 1.00 and 2.30, PPP between 40.00 and 314.67, TSPP between 48.55 and 364.71, and HSW between 8.53 and 39.50 g. Previously, DF in the range of 35–53 days and DM in the range of 88–110 days were reported across three chickpea cultivars grown in greenhouses [25]. The same study reported SPP, PPP, and HSW in the ranges of 0.9–1.1, 23–76, and 16.20–21.40 g, respectively. In another study, Seyedimoradi et al. [26] reported DF of 54.16 to 70.00 days, PPP of 3.40–17.00, TSPP of 3.13–20.00, and HSW of 29.74–41.11 g across 167 field-grown chickpea varieties. Other studies also reported such wide-ranging values [27,28,29].

In general, the obtained values in our study were comparable with many of the previous studies while others reported much higher and/or lower ranges. Such discrepancies could be attributed to the differences in growth conditions, number of accessions, genotype, and year of cultivation, among others [14,30,31]. Early maturing genotypes are highly desired for sustainable agriculture and breeding programs [32]. In this study, a total of 27.87% of the chickpea accessions were found to have a lower DM than the average (102.19 days) and hence, they could be important materials. Likewise, 50.82, 46.72, 50.00, and 41.80% of the accessions had higher SPP, PPP, TSPP, and HSW than the average values, respectively. Out of all these, two early maturing accessions, including breeding lines CP018 and CP019, simultaneously displayed SPP, PPP, TSPP, and HSW higher than the average values, making them superior accessions. Another eight breeding lines (CP118, CP017, CP021, CP026, CP022, CP066, CP037, CP015) and one landrace (CP109) with lower DM than the average also displayed higher SPP, PPP, and TSPP than the average values (Figure 1). These accessions could therefore be used in the development of early maturing and high-yielding chickpea varieties [14,18].

### 2.2. Variation of Crude Protein, Total Fat, Crude Fiber, and Dietary Fiber Contents

The crude protein, total fat, crude fiber, and dietary fiber contents across the 122 chickpea accessions are shown in Figure 1 and the summary data is displayed in Table 2.

With a CV of 19.89%, total fat content showed a wider variance than crude protein content (CV: 13.08%) and ranged from 2.54 to 6.19%. The crude protein content was in the range of 15.05–30.75%. Likewise, the crude fiber content showed approximately a two-fold variance (33.61%) compared to the dietary fiber content (17.25%). With means of 5.33 and 19.82%, the crude fiber and dietary fiber contents were in the ranges of 1.85–9.76 and 9.73–26.96%, respectively. Previously, protein content in the range of 16.10–21.30%, and fat content in the range of 3.10–5.50% were reported across six chickpea cultivars [33]. The same study reported a crude fiber content ranging from 4.6 to 12.70%. Another study conducted on 11 chickpea varieties reported a total fat content of 5.24–6.43%, a crude protein content of 16.34–19.18%, and a crude fiber content of 5.12–7.49% [34]. Other studies also reported wide-ranging values across different chickpea genotypes [35,36,37]. Compared to most of these previous studies, this study found wider variances in crude protein, total fat, crude fiber, and dietary fiber contents which could be attributed to the large sample size considered in addition to the environmental and genetic factors described before [13,14,15,16,17].

Chickpea is known for its protein content and has been utilized in food industries and augmented with other dietary crops [6,38]. In this study, approximately 55.00% and 39.00% of the chickpea accessions displayed higher crude protein content and total fat contents than the average values, respectively. Interestingly, out of those superior accessions with better yield traits, five breeding lines (CP026, CP037, CP022, CP021, and CP066) and landrace CP109 were included in this category. Moreover, fibers are known for having a variety of health benefits including reducing the risk of diabetes, hypertension, and gastrointestinal diseases among others [9]. Therefore, crops with maximized levels of fiber are desirable in food industries, breeding programs, and for daily consumption [7,8]. In this study, 44.26% of the total population simultaneously displayed higher crude fiber and dietary fiber contents than the average values. Once again, breeding lines CP037, CP022, CP021, and CP066 were grouped in this category. Furthermore, breeding line CP026 and landrace CP109 also displayed higher crude fiber and dietary fiber contents, respectively than the average values (Figure 1). Therefore, these accessions could be good sources of nutrition in addition to their yield performance [7,8,9]. The overall performance and general information of these superior accessions are summarized in Table S2.

### 2.3. Variation of Fatty Acid Contents

Chickpea is known to contain fatty acids with different oxidation states that have health benefits and hence, analyzing their diversity among different genetic materials is crucial [39,40,41]. This study found five major fatty acids including two saturated (palmitic acid and stearic acid) and three unsaturated (oleic acid, linoleic acid, and linolenic acid) fatty acids in all 122 chickpea accessions with wide ranges of contents (Figure 1). Palmitic and stearic acid contents were in the ranges of 7.29–9.79 and 0.83–1.63% with CVs of 6.89 and 15.59%, respectively. Likewise, the contents of oleic acid, linoleic acid, and linolenic acid were in the ranges of 13.32–69.22, 20.40–71.84, and 1.34–5.76% with CVs of 43.04, 13.69, and 22.80%, respectively (Table 3). In all accessions, palmitic acid was the dominant unsaturated fatty acid contributing more than 81.00% to the total unsaturated fatty acid content (TSFA). This observation agrees with previous observations in chickpea and other legumes [32,39,40]. Likewise, linoleic acid was the dominant unsaturated fatty acid contributing more than 50.00% to the total unsaturated fatty acid content (TUFA). Exceptions were landraces CP068, CP076, CP077, and CP067 as well as breeding line CP057 which displayed oleic acid as the dominant unsaturated fatty acid. Such variance could be attributed to the abundance of fatty acid desaturase (FAD) enzymes that govern the interconversion of fatty acids [41].

The presence and distribution of essential unsaturated fatty acids such as omega-6 (ω-6) and omega-3 (ω-3) fatty acids are desirable in legumes owing to their wide-ranging health benefits [11]. In this study, 74.59% of the accessions were found to have a higher linoleic acid content than the average (64.32%). Out of these, 31.87% of the accessions had a higher linolenic acid content than the average value (3.89%) and hence, could be sources of healthy lipids owing to their lower ω-6 to ω-3 ratio [38,39]. On the other hand, the abundance of unsaturated fatty acids could affect the oil stability of legumes and hence, much higher levels are not desirable. In this regard, those accessions with a lower content of unsaturated fatty acid could be important genetic resources [11].

### 2.4. Effect of Origin on Yield Traits and Nutritional Components

As highlighted before, different factors associated with genetics and environment affect the yield and overall quality of crops in general, including legumes. Studying the effects of such factors assists in selecting superior varieties that could be used in breeding, food industries, and for future conservation [15,18,42,43]. In this study, the effect of origin difference on the levels of all analyzed traits was statistically investigated. Table 4 summarizes the variations of yield traits and nutritional components among chickpea materials of different origins. Being the earliest to flower and mature, Indian accessions showed typical characteristics with regard to yield traits displaying the highest average values of all except for HSW. In contrast, Ukrainian accessions were late flowering and maturing exhibiting the lowest average SPP, PPP, and TSPP values. Interestingly, the variations between the highest and the lowest average values of all these traits were significantly different (*p* < 0.05). Studies that statistically investigated the variations of yield traits among chickpea genetic materials of different origins are scarce. However, some studies conducted within specific countries showed how geographical variation affects the agronomic traits of chickpea resources [44,45]. A recent genome-wide association study also showed the diversity in agronomic traits among chickpea materials collected from different countries [24]. Despite the differences in the number of accessions, the observed results signify that origin could be used as a parameter to categorize chickpea genetic materials based on their yield parameters.

Regarding the nutritional components, Indian accessions displayed the highest average total protein and crude fiber contents. Indian accessions also displayed the second-highest dietary fiber content and the lowest average total fat content (Table 4). In contrast, the lowest average total protein and crude fiber contents were found in Georgian accessions. Likewise, accessions from Bulgaria exhibited the highest average total fat content, while those from Myanmar displayed the highest average dietary fiber content. The lowest average dietary fiber content was found in Ukrainian accessions. Once again, the variations between the highest and lowest values were statistically significant (*p* < 0.05) signifying the effect of origin on the nutritional qualities of chickpea. Although not common in chickpea, previous studies conducted on soybean genetic materials reported contradicting results regarding the effect of origin. For instance, Azam et al. [46] and Lee et al. [47] found significant variations in total protein content among soybeans of different origins. In contrast, two other separate studies did not find such significant variations [48,49]. Once again, such discrepancies could be attributed to differences in genotype, growth condition, cultivation year, and postharvest handling processes [13,14,15]. The individual fatty acids also showed significant variations among the different countries of origin. In our previous study, origin difference showed a significant effect on all soybean fatty acids which corroborates the findings in this study [49]. In contrast, the total fatty acids contents, including TSFA and TUFA, did not show significant differences between any of the countries of origin. Typically, the variations observed in the levels of oleic acid and linoleic acid were noteworthy. Oleic acid content increased in the order of Bulgaria > Ukraine > Georgia > India > Myanmar while linoleic acid content showed the opposite trend. The fact that the levels of these two essential fatty acids varied among the different countries signifies that origin could be used to classify chickpea genetic materials according to their lipid quality [50].

### 2.5. Variations of Phenotypic Traits Among Chickpea Cultivars, Breeding Lines, and Landraces

This study also statistically investigated the variations of all analyzed phenotypic traits between the chickpea cultivars, breeding lines, and landraces to view the effect of genotype difference. Among the agronomic traits (Table 1), all the traits except for SPP showed significant variations (*p* < 0.05). The average DF and DM decreased in the order of cultivar > landrace > breeding line while DFM, PPP, and TSPP showed the opposite trend. In contrast, the average HSW was the highest in cultivars and the lowest in landraces (*p* < 0.05). Several studies showed the effect of genotype variations on yield traits in different types of legumes [32,51,52]. In chickpea, however, many of the studies focused on a specific type of genotype [25,27,28,29]. Other studies also considered chickpea materials of different statuses but failed to conduct statistical analysis to view the variations between genotypes [26,42]. In a more recent study, Misra et al. [21] showed significant variation in DF and HSW between desi and kabuli seed types of nineteen chickpea cultivars. Overall, the results of this study signified that yield traits showed significant variations among the different chickpea genotypes, and hence, could be used as a parameter for selecting varieties with desirable qualities [18,42,43].

The variations of crude protein, total fat, crude fiber, and dietary fiber contents between the different chickpea genotypes were also statistically analyzed (Table 2). Accordingly, breeding lines had the highest average protein, crude fiber, and dietary fiber contents. In contrast, landraces displayed the highest average total fat and the lowest average crude protein contents, while cultivars displayed the lowest average crude fiber, and dietary fiber contents (Table 2). Interestingly, the differences between the highest and lowest average values of all the parameters were statistically significant (*p* < 0.05). Although previous studies found significant variation in nutrient levels among different chickpea varieties, studies that investigate the effect of genotype differences on the levels of crude protein, total fat, crude fiber, and dietary fiber contents are scarce [21,53]. In general, the significant variations observed in this study on the levels of the nutritional components denote the importance of genotype in classifying chickpea genetic materials in accordance with their nutritional values [21,43,53]. Statistical analysis was also conducted to view the effect of genotype differences on the levels of fatty acids. Once again, all the fatty acids, except for palmitic acid, showed significant variations (Table 3). Specifically, the average stearic acid and oleic acid contents decreased in the order of cultivars > landraces > breeding lines while the average linoleic acid and linolenic acid contents showed the opposite trend. In a previous study, Gul et al. [16] found a significant effect of genotype variation on palmitic acid, oleic acid, linoleic acid, and linolenic acid. In another study Salaria et al. [13] reported a significant effect of genotype on palmitic acid and linolenic acid but not on linoleic acid and oleic acid. Such variable results have been documented in other legumes as well which could, once again, be attributed to the differences in growing conditions, analysis protocols, year of cultivation, and genotype [54,55,56]. In contrast to individual fatty acids, this study found no significant variation in the levels of total fatty acid contents among the chickpea genotypes (Figure S1). In general, the observed results signify that the different chickpea genetic materials could be categorized based on the levels of their fatty acids, particularly unsaturated fatty acids [12,13].

### 2.6. Multivariate Analysis

Multivariate statistical tools such as hierarchical cluster analysis (HCA), principal components analysis (PCA), and Pearson’s correlation analysis are important to further view the distributions of plant genetic materials and their association with phenotypic and genotypic traits [18,57]. The entire data set obtained for the 122 chickpea accessions was computed for HCA, PCA, and Pearson’s correlation analysis.

**Figure 2:**
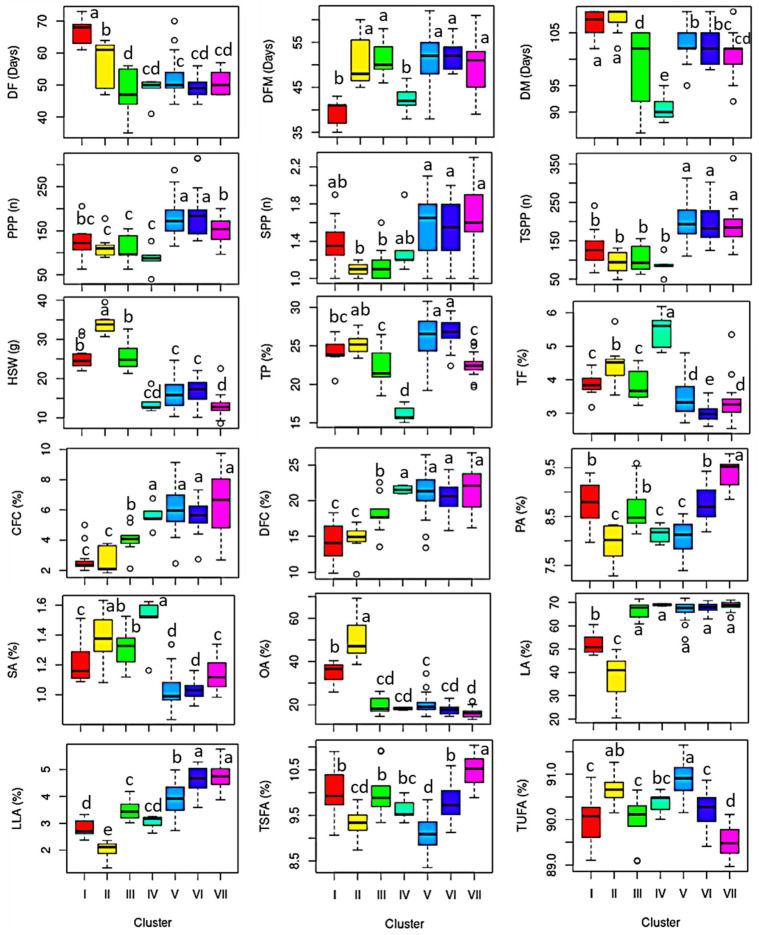
Variations of phenotypic traits across chickpea clusters obtained from hierarchical cluster analysis. Different letters on boxplots indicate significantly different means based on Duncan’s multiple range test (*p* < 0.05). CFC: Crude fiber content, DFM: Days from flowering to maturity, DF: Days to flowering, DFC: Dietary fiber content, DM: Days to maturity, HSW: One-hundred seeds weight, LA: Lin-oleic acid, LLA: Linolenic acid, OA: Oleic acid, PA: Palmitic acid, PPP: Number of pods per plant; TSPP: Total seeds per plant; SA: Stearic acid, SPP; Number of seeds per pod, TF: Total fat, TP: Total protein, TSFA: Total saturated fatty acid, TUFA: Total unsaturated fatty acid.

Figure 1 shows the HCA matrix which classified the chickpea accessions into seven clusters. The largest number of accessions was found in cluster V (n = 46) followed by clusters VI (n = 26), VII (n = 17), and I (n = 12). The other clusters including II, III, and IV contained 7, 9, and 5 accessions, respectively. Cluster IV exclusively contained Myanmar accessions. Indian accession dominated cluster VI accounting for 84.62%. Clusters V and VII contained a comparable number of accessions from these two countries, while clusters I, II, and V were the most heterogeneous containing accessions from at least three different countries (Table S1). Landraces dominated cluster IV (100%) while breeding lines dominated cluster VI (84.62%). Likewise, the majority of the cultivars were found in cluster I (n = 8). Statistical analysis showed that all the analyzed phenotypic traits showed significant variations among the different clusters (Figure 2 and Table S3). In summary, accessions in cluster VII showed characteristic features displaying the highest average SPP, crude fiber, dietary fiber, palmitic acid, linolenic acid, and TSFA. These accessions also displayed the second-highest average linoleic acid and the lowest HSW and oleic acid. In contrast, early maturing accessions with the lowest average DM were found in cluster IV and displayed the highest average stearic acid, linoleic acid, and total fat contents. Despite their early maturity, accession in cluster IV exhibited the lowest average PPP, TSPP, and total protein. In general, the HCA classified the chickpea accessions based on the performance of the phenotypic traits.

**Figure 3:**
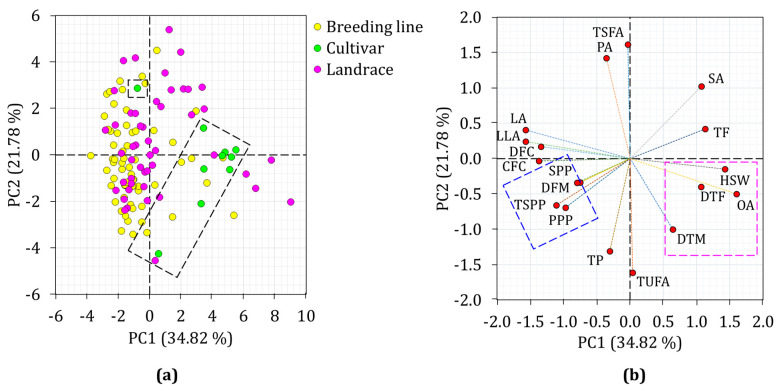
Score plot of chickpea accessions (**a**) and loading plot of variables (**b**) obtained from PCA. CFC: Crude fiber content, DFM: Days from flowering to maturity, DF: Days to flowering, DFC: Dietary fiber content, DM: Days to maturity, HSW: One-hundred seeds weight, LA: Linoleic acid, LLA: Linolenic acid, OA: Oleic acid, PA: Palmitic acid, PPP: Number of pods per plant; TSPP: Total seeds per plant; SA: Stearic acid, SPP; Number of seeds per pod, TF: Total fat, TP: Total protein, TSFA: Total saturated fatty acid, TUFA: Total unsaturated fatty acid.

PCA was also computed to further view the distribution of the chickpea accessions and their association with the analyzed parameters (Figure 3). The first five components had Eigenvalues greater than 1 explaining more than 80.00% of the total variance. Among these, the first two (PC1 and PC2) contributed 56.60% and the distribution of the chickpea accessions and their association with the analyzed parameters was computed along the axis of these components.

As shown in the score plot, all the cultivars, except one, tended to group themselves along the positive axis of PC1 (Figure 3a). In contrast, the majority of the breeding lines were on the opposite side, while landraces were widely dispersed along the PC1 and PC2 axes. Accessions from Ukraine were also clearly separated from those of India and Myanmar (Figure S2a). When the score plot was computed based on their cluster, the chickpea accessions were grouped supporting the HCA observation (Figure S2b). The loading plot showed a wide spread of the examined phenotypic traits along the two principal components and demonstrated different contributions to the observed variance (Figure 3b). Accordingly, PPP, DF, TSPP, HSW, total fat, crude fiber, dietary fiber, oleic acid, linoleic acid, and linolenic acid were the major contributors to the variance observed along PC1 with a factor loading of >±0.50, and contributions ranging from 4.54 to 12.32% (Table S4). Likewise, DM, total protein, palmitic acid, TSFA, and TUFA were the major factors for the variance observed along PC2 with contributions ranging from 7.71 to 20.03%. The loading plot also showed variable degrees of association between the variables. In particular, the associations of yield traits including SPP, PPP, and TSPP as well as DM, DF, HSW, and oleic acid were visible on the opposite sides of PC1 supporting the HCA observation.

**Figure 4:**
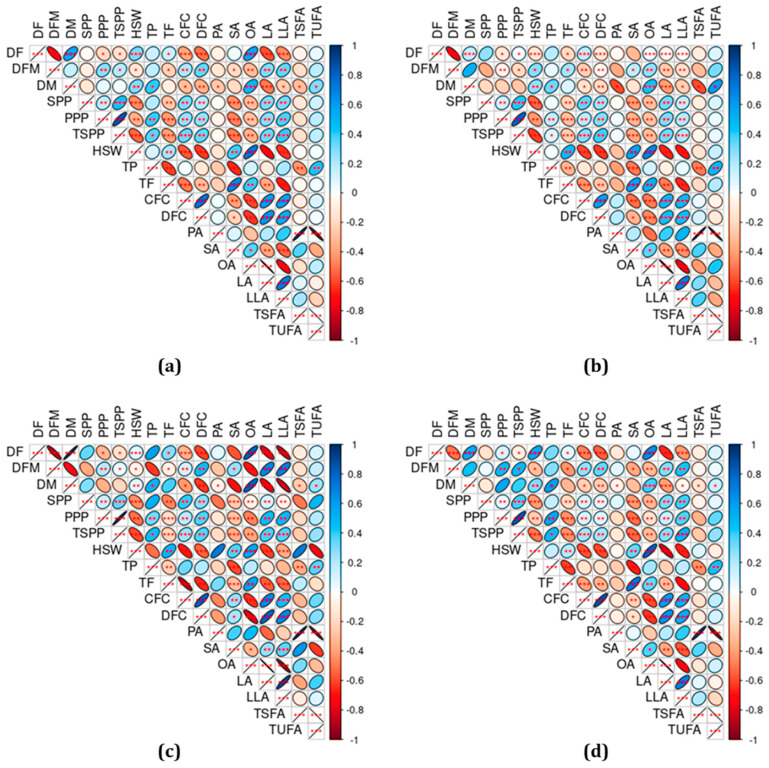
Pearson’s correlation matrix of yield traits and nutritional components in the whole population (**a**), cultivars (**b**), landraces (**c**), and breeding lines (**d**). CFC: Crude fiber content, DFM: Days from flowering to maturity, DF: Days to flowering, DFC: Dietary fiber content, DM: Days to maturity, HSW: One-hundred seeds weight, LA: Linoleic acid, LLA: Linolenic acid, OA: Oleic acid, PA: Palmitic acid, PPP: Number of pods per plant; TSPP: Total seeds per plant; SA: Stearic acid, SPP; Number of seeds per pod, TF: Total fat, TP: Total protein, TSFA: Total saturated fatty acid, TUFA: Total unsaturated fatty acid. * *p* < 0.05, ** *p* < 0.01, *** *p* < 0.001.

Pearson’s correlation analysis was computed to view the degree of association between the analyzed parameters and some noteworthy correlations were observed supporting the PCA observation (Figure 4). For instance, DF and DM showed a positive and significant correlation (*p* < 0.001) in the total population (Figure 4a) as well as in the different chickpea genotypes (Figure 4b–d). Similarly, SPP had a positive and significant correlation with PPP (*p* < 0.01) and TSPP (*p* < 0.001) regardless of genotype difference. PPP and TSPP also showed a significant correlation with each other across all the chickpea genotypes (*p* < 0.001). Such positive correlations between yield traits have been reported in previous studies and were used in selecting high-yielding varieties [28,29,30]. On the other hand, HSW showed a positive correlation with total fat, stearic acid, and oleic acid, and a negative correlation with linoleic acid and linolenic acid at different levels of significance across all the chickpea genotypes. DM also showed variable associations with the unsaturated fatty acids at different levels of significance. These observations could signify the effect of maturity period and seed weight on the levels of chickpea fatty acids which could be a research focus in future studies [16]. Crude fiber and dietary fiber also showed a positive and significant correlation with linoleic acid and linolenic acid regardless of genotype difference. The negative and significant association of oleic acid with linoleic acid and linolenic acid in all the chickpea genotypes further asserts the action of FAD enzymes [13,41].

## 3. Materials and Methods

### 3.1. Chemicals and Reagents

Analytical grade reagents and chemicals were used in this study. Sulfuric acid (H_2_SO_4_) and ethanol were obtained from Fisher Scientific (Pittsburgh, PA, USA). The remaining chemicals including hexane, heptane, benzene, acetone, petroleum ether, and fatty acid standards (palmitic acid, stearic acid, oleic acid, linoleic acid, and linolenic acid) were ordered from Sigma-Aldrich (St. Louis, MO, USA).

### 3.2. Plant Materials Collection, Cultivation, and Trait Recording

**Figure 5:**
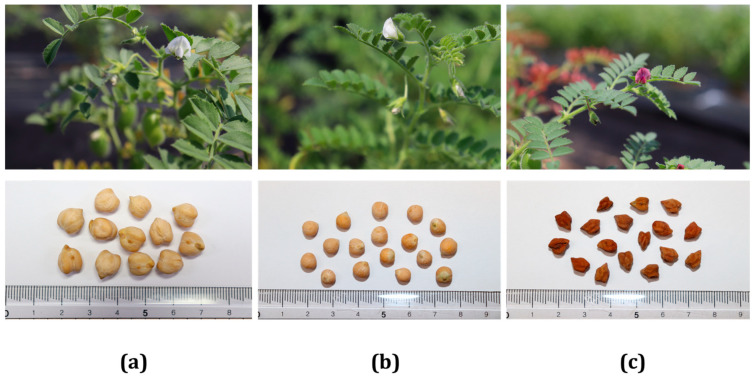
Representative flowers and seeds of chickpea cultivar (**a**), landrace (**b**), and breeding line (**c**) grown in a greenhouse.

The seeds of 122 chickpea accessions consisting of 10 cultivars, 48 landraces, and 64 breeding lines, were obtained from the genebank at the National Agrobiodiversity Center, Rural Development Administration (Jeonju, Republic of Korea). Cultivation was also conducted at the same center. For each accession, 25 seeds were sown on plug trays on 22 March 2022. After 20 days, 17 randomly selected seedlings from each accession were planted in rows on sandy loam soil in a greenhouse. The row plot was 80 × 20 cm, and the cultivation period lasted until July 2022. During this period, the cultivation condition was kept uniform for all the accessions. The growth traits including DF, DFM, and DM were documented from field inspection. Likewise, yield traits including PPP, SPP, and TSPP were recorded. HSW was recorded from laboratory examination after hand-harvest. In addition to their status (Figure 5), the chickpea materials were also grouped according to their origin: India (n = 65), Myanmar (n = 40), Ukraine (n = 8), Bulgaria (n = 7), and Georgia (n = 2). Accordingly, all materials from Myanmar and Georgia were landraces. In contrast, all materials from India, except for one cultivar, were breeding lines. Materials from Bulgaria contained 4 cultivars and 3 landraces, while those from Ukraine contained 5 cultivars and 3 landraces. Mature seed samples from each group were freeze-dried in an LP500 freeze dryer (ilShinBioBase, Dongducheon, Republic of Korea), powdered, sieved through 500 µm mesh size, and kept at −20 °C pending the analysis of nutritional components. General information regarding the 122 chickpea accessions including their temporary introduction number, code given (CP001-CP122), and genotype is provided in Table S1 (Supplementary Materials).

### 3.3. Analysis of Nutritional Components

#### 3.3.1. Crude Protein Content

For the analysis of crude protein content, an Auto-digester instrument that was preheated to 420 °C (FOSS, Hoganas, Sweden) was used and the Kjeldahl method was applied [58]. For each accession, 0.5 g of powdered seed sample was prepared in triplicate and put into separate digestion tubes. Then, 12 mL of concentrated H_2_SO_4_ along with two pellets of selenium catalyst was added to each and the mixtures were hydrolyzed for 1 h. After that, the digestion tubes were taken off, cooled at room temperature (~25 °C), and processed using a Kjeltec analyzer (FOSS, Hoganas, Sweden). The crude protein content was then calculated from the percent nitrogen content multiplied by 6.25 (standard Kjeldahl factor) from the triplicate measurements.

#### 3.3.2. Total Fat Content

Oil extraction was achieved by a soxhlet extraction method using an automated Soxtec instrument (FOSS, Tecator, Hoganas, Sweden). Once again, extraction and analysis were conducted in triplicate [59]. Specifically, 0.7 g of powdered seed sample was mixed with 50 mL of n-hexane. The boiling time was set to 30 min, while the rinsing and recovery times were set to 60 and 20 min, respectively. The total oil content, in percent, was determined as the weight of oil obtained per weight of extracted seed sample on a dry weight basis.

#### 3.3.3. Crude Fiber Content

The method of the Association of Official Analytical Chemists (AOAC) was applied to determine the crude fiber content using a Fiber Analyzer (FOSS, Hillerød, Denmark) [59]. Briefly, 0.7 g of powdered seed sample was sealed in filter bags and defatted in petroleum ether for 10 min. Then, the solvent was discarded and the filter bags were air-dried followed by 40 min of agitation in H_2_SO_4_ to extract crude fiber. The filter bags were removed and immersed in acetone and recovered after 10 min followed by drying (105 °C, 2 h) and cooling in a desiccator for 30 min. The filter bags were weighed, incinerated in an electric furnace (550 °C, 2 h), cooled for 40 min in a desiccator, and then re-weighed. Finally, the crude fiber content was calculated as a percentage of the loss in weight after incineration.

#### 3.3.4. Dietary Fiber Content

The dietary fiber content was analyzed using an Analytical Fibertec E-1023 System (FOSS, Hillerød, Denmark), as described previously [59]. Briefly, 1 g of powdered seed sample was suspended in 50 mL phosphate buffer (0.08 M). The mixture was digested using α-amylase (0.1 mL) and incubated at 100 °C for 15 min followed by successive digestion using protease and amyloglucosidase (0.1 mL each) at 60 °C for 30 min each, with proper pH adjustment. The mixture was then treated with four volumes of 95% ethanol, stirred, and then incubated overnight at ~25 °C. The obtained precipitate was filtered through a crucible with Celite, successively washed with 78% ethanol, 95% ethanol, and pure acetone. The precipitate was dried overnight at 60 °C and weighed to determine percent DFC.

#### 3.3.5. Fatty Acid Analysis Using Gas Chromatography Instrument

For fatty acid analysis, fatty acid methyl ester derivatives were synthesized using a direct methylation technique as reported in our recent study without modification [60]. The identification and contents of the fatty acids were conducted using a QP2010 gas chromatograph-flame ionization detector (GC-FID) instrument (Shimadzu, Kyoto, Japan). The column used was an HP-INNOWAX column (30 m × 0.250 mm, 0.25 µm) from Agilent Technologies (Santa Clara, CA, USA) and the injection volume was 1 µL at a split ratio of 50:1. The carrier gas was helium and its flow rate was maintained at a rate of 1.5 mL/min. During analysis, the initial column temperature started at 100 °C and increased to 170 °C at a rate of 60 °C/min with a holding time of 1 min. It was then raised to 240 °C at a rate of 6.5 °C/min and held for another 1 min taking a total run time of 16.4 min. The temperature of the detector and injection port was set at 250 °C. The acquired chromatogram was analyzed using LabSolution software version 5.92 (Shimadzu, Kyoto, Japan). The individual fatty acids were identified using the retention times of the corresponding external standards. The contents of individual fatty acids were calculated using peak percentage.

### 3.4. Statistical Analysis

In this study, all measurements and analyses were conducted in triplicates. Results are expressed as mean ± standard deviation (SD). Analysis of variance (ANOVA) followed by Duncan’s multiple-range test) was applied to statistically determine significant differences between measurements using XLSTAT software version 2019.2.2 (Lumivero, Denver, CO, USA). A two-way hierarchical cluster analysis (HCA) and Principal component analysis (PCA) were performed using JMP software version-17 (SAS, Inc., Cary, NC, USA). Pearson’s correlation analysis was computed using R-software version 4.0.2 (r-project).

## 4. Conclusions

Phenotypic traits in crops, including agronomical and biochemical parameters, are affected by different factors related to environment and genetic variances. Studies that investigate the effects of these factors are highly required in breeding as well as in selecting varieties that have desirable qualities. This study showed the diversity of key yield traits and nutritional components among chickpea breeding lines, landraces, and cultivars collected from five different countries and grown in a greenhouse. All the analyzed parameters showed significant variations among the chickpea genetic materials. Among all the studied materials, five breeding lines from India and one landrace from Myanmar were identified as superior accessions. These accessions excelled in both yield-related traits and nutritional parameters. Therefore, they could be ideal candidates for breeding programs, dissemination to local farmers, and conservation. It would also be beneficial to investigate their growth performances in the Korean agricultural environment. Moreover, origin and genotype differences demonstrated significant effects on most of the yield traits as well as nutritional components. Although this study considered only the major phenotypic traits, the results obtained signify the importance of considering origin and genotype when selecting chickpea genetic materials for yield performance and nutritional value. Future research should focus on comprehensive metabolomics and genomics studies as well as exploring the effect of genotype-environment interaction on chickpea metabolites and agronomic traits.

## Figures and Tables

**Table 1:** Variations of quantitative agronomic traits among chickpea breeding lines, cultivars, and landraces.

Trait	Value	Genotypes			Total
		Breeding Line	Cultivar	Landrace	
Days to flowering (Days)	Minimum	44.00	37.00	35.00	35.00
	Maximum	64.00	73.00	64.00	73.00
	Mean	50.23 ^c^	66.20 ^a^	52.75 ^b^	52.53
	SD	4.11	9.94	5.70	6.92
	CV	8.19	15.01	10.81	13.17
Days from flowering to maturity (Days)	Minimum	38.00	35.00	38.00	35.00
	Maximum	61.00	49.00	62.00	62.00
	Mean	51.27 ^a^	39.70 ^b^	49.58 ^a^	49.66
	SD	4.75	3.82	5.33	5.80
	CV	9.27	9.63	10.74	11.69
Days to maturity (Days)	Minimum	92.00	86.00	88.00	86.00
	Maximum	109.00	109.00	109.00	109.00
	Mean	101.50 ^b^	105.90 ^a^	102.33 ^b^	102.19
	SD	3.11	6.82	5.49	4.71
	CV	3.07	6.44	5.37	4.61
Seeds per pod (n)	Minimum	1.00	1.00	1.00	1.00
	Maximum	2.30	1.90	2.00	2.30
	Mean	1.55 ^a^	1.38 ^a^	1.41 ^a^	1.48
	SD	0.33	0.30	0.29	0.32
	CV	21.31	21.69	20.15	21.51
Pods per plant (n)	Minimum	90.00	63.33	40.00	40.00
	Maximum	314.67	205.67	260.67	314.67
	Mean	170.15 ^a^	127.55 ^b^	145.28 ^b^	156.87
	SD	47.50	38.86	40.63	46.61
	CV	27.92	30.47	27.96	29.71
Total seeds per plant (n)	Minimum	65.71	66.53	48.55	48.55
	Maximum	364.71	241.38	313.27	364.71
	Mean	186.62 ^a^	143.94 ^b^	158.44 ^b^	172.04
	SD	58.00	52.48	58.95	60.06
	CV	31.08	36.46	37.21	34.91
One-hundred seeds weight (g)	Minimum	9.50	17.63	8.53	8.53
	Maximum	35.07	26.47	39.50	39.50
	Mean	18.37 ^b^	23.78 ^a^	17.34 ^b^	18.41
	SD	5.73	2.34	7.11	6.35
	CV	31.17	9.84	40.98	34.50

Different superscript letters in a row represent significantly different means at *p* < 0.05.

**Table 2:** Variations of total nutritional contents among chickpea breeding lines, cultivars, and landraces.

Nutritional Component	Value	Genotypes			Total
		Breeding Line	Cultivar	Landrace	
Total protein (%)	Minimum	20.96	21.18	15.05	15.05
	Maximum	30.78	29.32	29.61	30.78
	Mean	25.97 ^a^	24.88 ^ab^	23.08 ^b^	24.74
	SD	2.29	2.12	3.73	3.24
	CV	8.83	8.54	16.15	13.08
Total fat (%)	Minimum	2.54	3.05	2.68	2.54
	Maximum	4.57	4.44	6.19	6.19
	Mean	3.24 ^b^	3.71 ^a^	3.94 ^a^	3.55
	SD	0.42	0.43	0.84	0.71
	CV	12.92	11.46	21.43	19.89
Crude fiber (%)	Minimum	3.49	2.30	1.85	1.85
	Maximum	9.76	6.82	9.13	9.76
	Mean	5.92 ^a^	3.61 ^c^	4.89 ^b^	5.33
	SD	1.41	1.56	1.92	1.79
	CV	23.85	43.25	39.31	33.61
Dietary fiber (%)	Minimum	13.43	11.94	9.73	9.73
	Maximum	25.46	22.56	26.69	26.69
	Mean	20.20 ^a^	15.61 ^b^	20.19 ^a^	19.82
	SD	2.81	3.79	3.49	3.42
	CV	13.94	24.25	17.27	17.25

Different superscript letters in a row represent significantly different means at *p* < 0.05.

**Table 3:** Variations of fatty acid contents among chickpea cultivars, landraces, and breeding lines.

Fatty Acid	Value	Genotypes			Total
		Breeding Line	Cultivar	Landrace	
Palmitic acid (%)	Minimum	7.29	7.39	7.40	7.29
	Maximum	9.69	9.14	9.79	9.79
	Mean	8.53 ^a^	8.46 ^a^	8.48 ^a^	8.51
	SD	0.60	0.47	0.58	0.59
	CV	7.09	5.61	6.83	6.89
Stearic acid (%)	Minimum	0.83	0.96	0.90	0.83
	Maximum	1.46	1.53	1.63	1.63
	Mean	1.06 ^b^	1.21 ^a^	1.19 ^a^	1.12
	SD	0.13	0.16	0.19	0.17
	CV	12.58	13.33	16.13	15.59
Oleic acid (%)	Minimum	13.32	14.72	14.45	13.32
	Maximum	46.77	40.39	69.22	69.22
	Mean	20.11 ^b^	34.03 ^a^	22.42 ^b^	22.16
	SD	6.02	7.54	11.66	9.54
	CV	29.95	22.15	52.02	43.04
Linoleic acid (%)	Minimum	42.41	47.48	20.40	20.40
	Maximum	70.98	71.48	71.84	71.84
	Mean	66.05 ^a^	53.49 ^b^	64.25 ^a^	64.32
	SD	5.22	7.34	11.00	8.80
	CV	7.90	13.73	17.12	13.69
Linolenic acid (%)	Minimum	2.09	2.38	1.34	1.34
	Maximum	5.76	3.92	5.39	5.76
	Mean	4.24 ^a^	2.81 ^c^	3.66 ^b^	3.89
	SD	0.78	0.42	0.83	0.89
	CV	18.44	14.82	22.71	22.80

Different superscript letters in a row represent significantly different means at *p* < 0.05.

**Table 4:** Variations of yield traits and nutritional components among chickpea materials of different origins.

Trait	Origin				
	Bulgaria	India	Ukraine	Myanmar	Georgia
Days to flowering (Days)	66.29 ± 3.15 ^a^	50.03 ± 4.40 ^b^	66.63 ± 4.77 ^a^	50.85 ± 4.12 ^b^	63.00 ± 1.00 ^a^
Days from flowering to maturity (Days)	41.57 ± 4.27 ^b^	51.23 ± 4.72 ^a^	40.63 ± 3.50 ^b^	50.68 ± 5.05 ^a^	42.50 ± 0.50 ^b^
Days to maturity (Days)	107.86 ± 1.81 ^a^	101.26 ± 3.63 ^b^	107.25 ± 3.03 ^a^	101.53 ± 5.53 ^b^	105.50 ± 0.50 ^ab^
Seeds per pod (n)	1.41 ± 0.34 ^ab^	1.54 ± 0.33 ^a^	1.28 ± 0.19 ^b^	1.45 ± 0.29 ^ab^	1.35 ± 0.05 ^ab^
Pods per plant (n)	149.38 ± 31.66 ^ab^	169.91 ± 47.18 ^a^	108.42 ± 25.94 ^b^	148.58 ± 42.48 ^ab^	119.33 ± 4.67 ^ab^
Total seeds per plant (n)	154.35 ± 58.34 ^ab^	186.14 ± 57.68 ^a^	108.17 ± 25.63 ^b^	168.09 ± 58.84 ^a^	109.82 ± 7.63 ^ab^
One-hundred seeds weight (g)	27.55 ± 6.82 ^a^	18.45 ± 5.71 ^b^	26.60 ± 3.62 ^a^	14.69 ± 3.53 ^c^	26.97 ± 5.00 ^a^
Total protein (%)	25.70 ± 1.89 ^ab^	25.90 ± 2.35 ^a^	24.30 ± 0.73 ^ab^	22.92 ± 4.01 ^ab^	22.07 ± 1.65 ^b^
Total fat (%)	4.15 ± 0.84 ^a^	3.24 ± 0.41 ^b^	4.00 ± 0.37 ^ab^	3.85 ± 0.86 ^ab^	3.87 ± 0.10 ^ab^
Crude fiber (%)	3.23 ± 1.77 ^b^	5.92 ± 1.40 ^a^	2.54 ± 0.64 ^b^	5.45 ± 1.60 ^a^	2.19 ± 0.03 ^b^
Dietary fiber (%)	15.86 ± 3.51 ^c^	20.24 ± 2.81 ^ab^	13.29 ± 2.15 ^c^	21.30 ± 2.35 ^a^	16.65 ± 0.24 ^bc^
Palmitic acid (%)	8.15 ± 0.61 ^b^	8.53 ± 0.60 ^ab^	8.66 ± 0.34 ^ab^	8.46 ± 0.56 ^ab^	9.25 ± 0.11 ^a^
Stearic acid (%)	1.25 ± 0.20 ^a^	1.06 ± 0.14 ^b^	1.27 ± 0.17 ^a^	1.16 ± 0.18 ^a^	1.14 ± 0.05 ^ab^
Oleic acid (%)	43.31 ± 13.48 ^a^	20.03 ± 6.02 ^b^	39.95 ± 8.16 ^a^	18.11 ± 2.61 ^b^	27.40 ± 1.56 ^b^
Linoleic acid (%)	44.85 ± 13.01 ^c^	66.14 ± 5.22 ^b^	47.54 ± 7.60 ^c^	68.39 ± 2.20 ^a^	58.94 ± 1.64 ^b^
Linolenic acid (%)	2.43 ± 0.53 ^c^	4.24 ± 0.78 ^a^	2.59 ± 0.45 ^c^	3.89 ± 0.64 ^ab^	3.27 ± 0.03 ^bc^
Total saturated fatty acid (%)	9.40 ± 0.64 ^a^	9.59 ± 0.64 ^a^	9.93 ± 0.39 ^a^	9.62 ± 0.61 ^a^	10.39 ± 0.05 ^a^
Total unsaturated fatty acid (%)	90.60 ± 0.64 ^a^	90.41 ± 0.64 ^a^	90.07 ± 0.39 ^a^	90.38 ± 0.61 ^a^	89.61 ± 0.05 ^a^

Different superscript letters in a row represent significantly different means at *p* < 0.05.

## Data Availability

All the data related to this study are incorporated in the manuscript and Supplementary Materials. Further inquiries can be directed to the first author.

## Supplementary Materials

The following supporting information can be downloaded at: https://www.mdpi.com/article/10.3390/plants13213078/s1, Figure S1: Variation of total fatty acid contents among different chickpea genotypes. Figure S2: Score plot of chickpea accessions obtained from PCA based on their origin (a) and cluster (b); Table S1: General information of greenhouse grown 122 chickpea accessions; Table S2: Properties and performances of selected superior chickpea accessions. Table S3: Variations of phenotypic traits across different clusters obtained from hierarchical cluster analysis. Table S4: Factor loadings and contributions of variables in the first five principal components.
