# Supplementary material for: Diversity of Major Yield Traits and Nutritional Components Among Greenhouse Grown Chickpea (Cicer arietinum L.) Breeding Lines, Landraces, and Cultivars of Different Origins
Source: Plants (Basel). 2024 Nov 1;13(21):3078. doi: 10.3390/plants13213078 (PMC11548202; doi:10.3390/plants13213078)
Supplement: Supplementary file 1 [file plants-13-03078-s001.zip › plants-3278246-supplementary.pdf]

Supplementary materials

Diversity of Major Yield Traits and Nutritional Components among Greenhouse Grown Chickpea (*Cicer arietinum* L.) Breeding Lines, Landraces, and Cultivars of Different Origins

Yu-Mi Choi [1], Hyemyeong Yoon [1], Myoung-Jae Shin [1], Sukyeung Lee [2], Jungyoon Yi [1], Xiaohan Wang [1] and Kebede Taye Desta [1,*]

[1] National Agrobiodiversity Center, National Institute of Agricultural Sciences, Rural Development Administration, Jeonju 54874, Republic of Korea.

[2] International Technology Cooperation Center, Technology Cooperation Bureau, Rural Development Administration, Jeonju 54875, Republic of Korea.

* Correspondence: kebedetdesta@korea.kr (K.T.D)

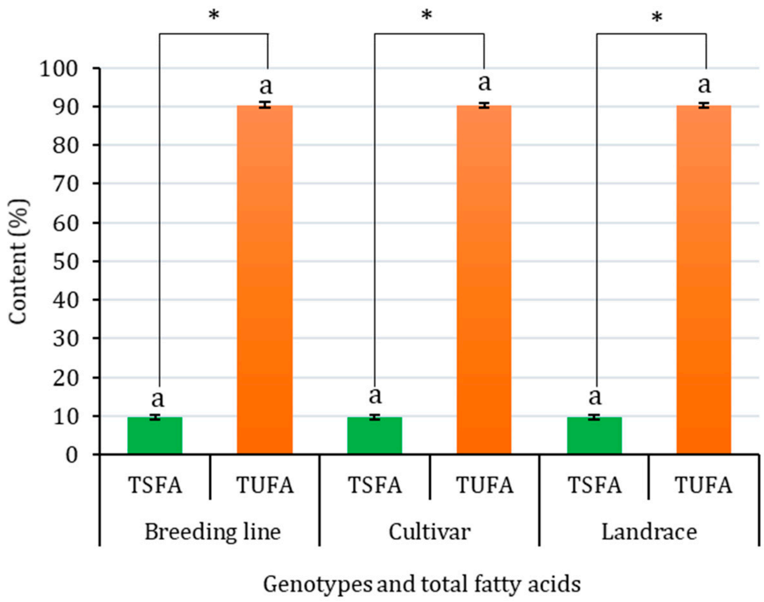

Figure S1. Variation of total fatty acid contents among different chickpea genotypes. Different letters on bars indicate significantly different means ($p < 0.05$). *$p < 0.05$. TSFA: Total saturated fatty acid, TUFA: Total unsaturated fatty acid.

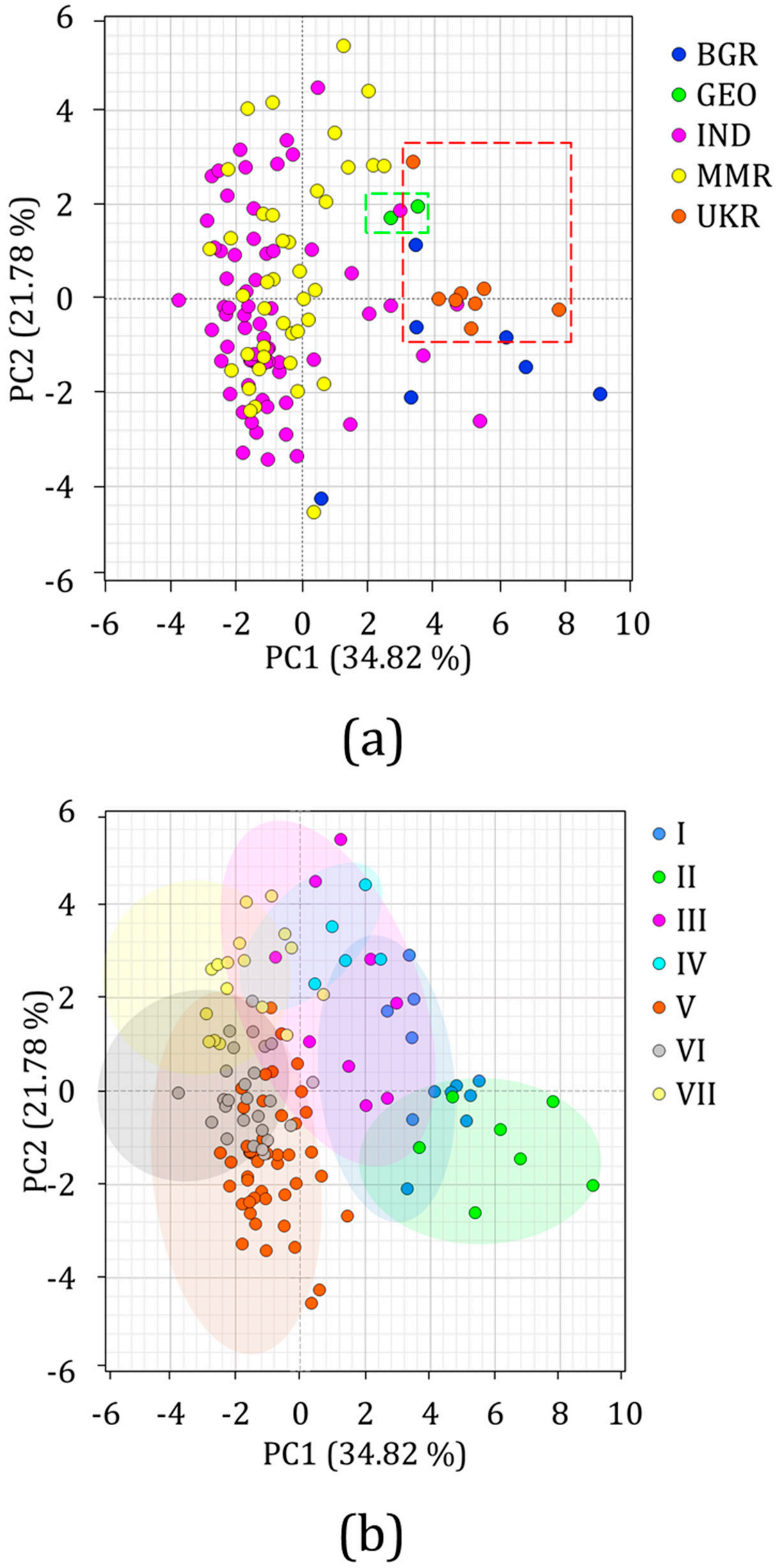

Figure S2. Score plot of chickpea accessions obtained from PCA based on their origin (a) and cluster (b). BGR: Bulgaria, GEO: Georgia, IND: India, MMR: Myanmar, UKR: Ukraine.

Table S1. General information of greenhouse grown 122 chickpea accessions.

Temp. number	Accession name	Code	Origin	Cluster number	Genotype	Temp. number	Accession name	Code	Genotype	Origin	Cluster number
K142005	DOBRUDZHANSKIJ 6	CP001	BGR	I	Cultivar	K142044	ICCL-80056	CP062	Breeding line	IND	V
K141999	OBRAZTZOV TCHIFLIK 1	CP002	BGR	I	Cultivar	K158096	ICC-11529	CP063	Breeding line	IND	VI
K142017	OBRAZTZOV TCHIFLIK 3	CP003	BGR	V	Cultivar	K158122	CSG-8962	CP064	Breeding line	IND	VII
K142007	PLOVDIV 8	CP004	BGR	I	Cultivar	K158095	Annegeri	CP065	Breeding line	IND	VII
K151229	ICC-482	CP005	IND	V	Breeding line	K158117	ICCL-80041	CP066	Breeding line	IND	VII
K142037	ICCL-81823	CP006	IND	V	Breeding line	K133761	A8E0493	CP067	Landrace	BGR	II
K142053	ICCL-80052	CP007	IND	VI	Breeding line	K133757	A8E0544	CP068	Landrace	BGR	II
K142027	ICC-1931	CP008	IND	V	Breeding line	K142003	Panskyi	CP069	Landrace	UKR	I
K142038	ICC-5003	CP009	IND	V	Breeding line	K142002	Kolorit	CP070	Cultivar	UKR	I
K142046	ICCL-81237	CP010	IND	VI	Breeding line	K142000	Fagot	CP071	Cultivar	UKR	I
K142022	ICCL-80046	CP011	IND	III	Breeding line	K141998	Smachnyi	CP072	Cultivar	UKR	I
K158090	ICC-11324	CP012	IND	V	Breeding line	K141997	Dobrobut	CP073	Cultivar	UKR	I
K142050	ICCC-36	CP013	IND	V	Breeding line	K142004	Ornament	CP074	Cultivar	UKR	I
K142054	ICC-11524	CP014	IND	VI	Breeding line	K142008	08UKR-166	CP075	Landrace	UKR	I
K158089	ICCL-80072	CP015	IND	VII	Breeding line	K133758	08UKR-327	CP076	Landrace	UKR	II
K158099	ICCl-80036	CP016	IND	V	Breeding line	K133759	A9E0121	CP077	Landrace	BGR	II
K158102	ICCL-4918	CP017	IND	VI	Breeding line	K158118	RAK-64	CP078	Landrace	MMR	V
K142021	ICCC-38	CP018	IND	VII	Breeding line	K158124	RAK-95	CP079	Landrace	MMR	V
K151000	ICCC-9	CP019	IND	VI	Breeding line	K158121	Leik Thei	CP080	Landrace	MMR	V
K158097	ICCL-80057	CP020	IND	III	Breeding line	K158101	ICCL-84218	CP081	Landrace	MMR	VII
K158087	ICCL-80045	CP021	IND	VI	Breeding line	K158106	JG-62	CP082	Landrace	MMR	V
K158115	ICCL-81220	CP022	IND	VI	Breeding line	K158108	JG-62 x Radhey	CP083	Landrace	MMR	VI
K142060	ICCL-4951	CP023	IND	VI	Breeding line	K158126	Kalape Alone Gyi	CP084	Landrace	MMR	V
K146844	ICCL-80034	CP024	IND	V	Breeding line	K158109	Karachi	CP085	Landrace	MMR	VII
K142034	ICCC-14302	CP025	IND	VI	Breeding line	K142062	Ah Phyu (Lone Thay)	CP086	Landrace	MMR	VII

K142025	ICCL-82110	CP026	IND	V	Breeding line	K142040	ICCL-86318	CP087	Landrace	MMR	V
K151013	ICCL-78026	CP027	IND	VI	Breeding line	K142042	ICCL-82211	CP088	Landrace	MMR	V
K142001	ICC-5676-K-4-1	CP028	IND	II	Breeding line	K142056	ICCL82223	CP089	Landrace	MMR	V
K158103	ICCL-79002	CP029	IND	V	Breeding line	K142023	RAK-19	CP090	Landrace	MMR	V
K158110	ICCL-80055	CP030	IND	VI	Breeding line	K142035	RAK-38	CP091	Landrace	MMR	V
K142041	ICC-12254	CP031	IND	V	Breeding line	K142059	RAK-22	CP092	Landrace	MMR	VI
K158125	ICCC-35	CP032	IND	VI	Breeding line	K142052	RAK-7 (Shwe Ni)	CP093	Landrace	MMR	V
K159277	ICCL-82202	CP033	IND	VI	Breeding line	K142010	Kalape Phyu Lay	CP094	Landrace	MMR	V
K195808	ICCL-12333	CP034	IND	VI	Breeding line	K142020	Madaya	CP095	Landrace	MMR	VI
K156141	ICCL-82208	CP035	IND	V	Breeding line	K142043	Ah Phyu (Lone Thay)	CP096	Landrace	MMR	VI
K142015	ICC-4948	CP036	IND	III	Breeding line	K142055	Kalape Lone Gyi	CP097	Landrace	MMR	V
K142061	ICC-11320	CP037	IND	VI	Breeding line	K151015	ICCV-790160-152P	CP098	Landrace	MMR	VII
K158104	ICCL-81001	CP038	IND	VI	Breeding line	K151012	ICCX-810713-104P	CP099	Landrace	MMR	VII
K142014	ICCL-82207	CP039	IND	V	Breeding line	K158123	Kalape	CP100	Landrace	MMR	V
K142006	ICCC-10450	CP040	IND	V	Breeding line	K158092	ICCL-8611	CP101	Landrace	MMR	V
K142030	ICC-1870	CP041	IND	V	Breeding line	K226630	ICCL-85108	CP102	Landrace	MMR	V
K142032	ICCC-82225	CP042	IND	V	Breeding line	K158120	ICCL-873220	CP103	Landrace	MMR	VII
K142029	H-208-T3	CP043	IND	VI	Breeding line	K272164	Kalape (Sagaing)	CP104	Landrace	MMR	V
K142036	ICCL-80063	CP044	IND	VI	Breeding line	K158119	CA - 86 - 61	CP105	Landrace	MMR	III
K142018	GS-269	CP045	IND	VI	Breeding line	K158091	CA - 86 - 23	CP106	Landrace	MMR	V
K142031	ICCL-79039	CP046	IND	VI	Breeding line	K226635	ICC - 16152	CP107	Landrace	MMR	V
K151112	ICC-11323	CP047	IND	V	Breeding line	K142033	ICC - 16227	CP108	Landrace	MMR	VII
K158112	ICCL-79010	CP048	IND	VII	Breeding line	K158100	ICC - 16153	CP109	Landrace	MMR	V
K158094	ICCL-14880	CP049	IND	III	Breeding line	K151023	ICC - 16144	CP110	Landrace	MMR	V
K158116	ICC-4458	CP050	IND	VII	Breeding line	K158113	ICC - 16198	CP111	Landrace	MMR	V
K142045	ICC-1881	CP051	IND	VII	Breeding line	K158107	ICC - 16181	CP112	Landrace	MMR	IV
K146794	ICCL-82101	CP052	IND	VI	Breeding line	K158093	ICC - 16132	CP113	Landrace	MMR	IV
K142051	ICCL-80044	CP053	IND	V	Breeding line	K158098	CA - 86 -105	CP114	Landrace	MMR	IV
K226634	ICC-4948 (G-130)	CP054	IND	VII	Breeding line	K158111	ICC - 16125	CP115	Landrace	MMR	IV

K142013	ICC-8346	CP055	IND	III	Breeding line	K151016	Ka Lar Pe	CP116	Landrace	MMR	IV
K142024	GS-290	CP056	IND	V	Breeding line	K142057	MMR-KOPIA KJT-2012-13	CP117	Landrace	MMR	III
K141995	ICC-4958	CP057	IND	II	Breeding line	K142019	IND-GJH-2013-11	CP118	Breeding line	IND	VII
K141996	C-44	CP058	IND	II	Breeding line	K142039	JG11	CP119	Breeding line	IND	III
K142028	ICC-10136	CP059	IND	V	Breeding line	K268447	JG14	CP120	Cultivar	IND	III
K142016	GS-136	CP060	IND	V	Breeding line	K142012	GEO-MKH-2018-91	CP121	Landrace	GEO	I
K158088	ICCL-80037	CP061	IND	V	Breeding line	K142009	GEO-HDY-2019-161	CP122	Landrace	GEO	I

BGR: Bulgaria, GEO: Georgia, IND: India, MMR: Myanmar, UKR: Ukraine.

Table S2. Properties and performances of selected superior chickpea accessions.

**Parameter/ property**	**Accessions**					
	CP021	CP022	CP026	CP037	CP066	CP109
Name	ICCL-80045	ICCL-81220	ICCL-82110	ICC-11320	ICCL-80041	ICC - 16153
Origin	India	India	India	India	India	Myanmar
Status	Breeding line	Breeding line	Breeding line	Breeding line	Breeding line	Landrace
Days to flowering (Days)	49.00	47.00	50.00	47.00	56.00	51.00
Days to maturity (Days)	98.00	99.00	100.00	99.00	97.00	99.00
Seeds per pod (n)	1.80 ± 0.40	2.00 ± 0.00	1.80 ± 0.40	1.50 ± 0.50	1.50 ± 0.50	2.00 ± 0.00
Pods per plant (n)	314.67 ± 57.74	201.67 ± 29.58	200.00 ± 17.45	202.33 ± 14.20	164.33 ± 21.14	158.00 ± 8.04
One hundred seeds weight (g)	17.00 ± 0.08	14.73 ± 0.17	16.43 ± 0.19	17.23 ± 0.26	9.50 ± 0.00	13.13 ± 0.12
Total protein (%)	25.89 ± 0.08	26.87 ± 0.10	27.33 ± 0.09	27.09 ± 0.05	25.51 ± 0.16	26.41 ± 0.06
Total fat (%)	3.18 ± 0.02	3.13 ± 0.02	3.32 ± 0.03	3.16 ± 0.04	3.01 ± 0.07	3.45 ± 0.02
Crude fiber (%)	7.24 ± 0.16	5.69 ± 0.05	6.82 ± 0.08	5.63 ± 0.14	7.70 ± 0.06	4.28 ± 0.02
Dietary fiber (%)	20.94 ± 0.17	21.05 ± 1.35	19.06 ± 0.66	20.63 ± 0.33	21.77 ± 0.66	21.66 ± 0.29
Palmitic acid (%)	8.61 ± 0.13	8.71 ± 0.17	7.70 ± 0.04	8.54 ± 0.10	9.69 ± 0.00	7.89 ± 0.01
Stearic acid (%)	1.08 ± 0.01	1.06 ± 0.02	0.83 ± 0.02	1.11 ± 0.02	1.05 ± 0.01	1.02 ± 0.00
Oleic acid (%)	17.57 ± 2.05	16.17 ± 1.98	22.29 ± 0.02	17.82 ± 0.06	13.96 ± 0.03	17.48 ± 0.02
Linoleic acid (%)	68.46 ± 1.96	69.81 ± 1.85	64.83 ± 0.06	67.96 ± 0.16	69.99 ± 0.02	69.20 ± 0.03
Linolenic acid (%)	4.28 ± 0.03	4.25 ± 0.05	4.35 ± 0.01	4.57 ± 0.02	5.31 ± 0.02	4.41 ± 0.00
TSFA	9.70 ± 0.14	9.77 ± 0.17	8.53 ± 0.06	9.65 ± 0.12	10.74 ± 0.01	8.91 ± 0.01
TUFA	90.30 ± 0.13	90.23 ± 0.17	91.47 ± 0.06	90.35 ± 0.12	89.26 ± 0.01	91.09 ± 0.01

TSFA: Total saturated fatty acid, TUFA: Total unsaturated fatty acid.

Table S3: Variations of phenotypic traits across different clusters obtained from hierarchical cluster analysis.

Trait	Clusters						
	**I**	**II**	**III**	**IV**	**V**	**VI**	**VII**
Days to flowering (Days)	66.92±3.99 a	56.43±7.13 b	47.11±7.43 d	48.40±3.77 cd	51.85±4.70 c	49.73±3.30 cd	51.00±3.60 cd
Days from flowering to maturity (Days)	39.67±2.56 b	51.00±5.50 a	51.22±3.61 a	42.40±3.01 b	51.24±4.91 a	52.15±2.73 a	49.35±5.84 a
Days to maturity (Days)	106.58±2.66 a	107.43±2.61 a	98.33±6.99 d	90.80±2.48 e	103.09±2.76 b	101.88±2.79 bc	100.35±3.77 cd
Seeds per pod (n)	1.38±0.24 ab	1.10±0.08 b	1.17±0.18 b	1.34±0.29 ab	1.56±0.31 a	1.55±0.27 a	1.62±0.34 a
Pods per plant (n)	123.64±33.77 bc	114.62±27.91 c	112.91±27.72 c	86.00±27.72 c	176.60±35.67 a	181.88±47.98 a	150.23±27.64 b
Total seeds per plant (n)	131.02±45.18 b	93.68±28.73 b	104.56±30.81 b	86.62±24.72 b	197.40±43.98 a	196.05±50.14 a	188.76±56.30 a
One hundred seeds weight (g)	25.39±3.02 b	34.05±2.75 a	25.62±3.45 b	13.95±2.47 cd	16.19±3.58 c	16.87±2.79 c	12.93±3.12 d
Total protein (%)	24.19±1.55 bc	25.23±1.32 ab	22.25±2.39 c	16.19±0.99 d	25.86±2.94 a	26.81±1.59 a	22.59±1.63 c
Total fat (%)	3.86±0.31 c	4.47±0.65 b	3.86±0.46 c	5.47±0.51 a	3.45±0.48 d	3.01±0.24 e	3.34±0.61 d
Crude fiber (%)	2.71±0.87 c	2.73±0.84 c	4.07±0.90 b	5.60±0.74 a	6.02±1.23 a	5.64±0.98 a	6.50±2.19 a
Dietary fiber (%)	14.30±2.47 c	14.53±2.17 c	18.11±2.53 b	21.55±0.52 a	21.17±2.46 a	20.53±1.86 a	21.57±2.88 a
Palmitic acid (%)	8.78±0.41 b	7.95±0.40 c	8.69±0.50 b	8.14±0.17 c	8.06±0.31 c	8.76±0.35 b	9.37±0.28 a
Stearic acid (%)	1.21±0.13 c	1.38±0.17 ab	1.32±0.12 b	1.49±0.17 a	1.03±0.11 d	1.03±0.06 d	1.138±0.10 c
Oleic acid (%)	35.14±4.39 b	50.91±10.05 a	19.80±3.75 cd	18.32±0.59 cd	19.90±3.53 c	17.77±2.16 cd	16.41±2.54 d
Linoleic acid (%)	52.06±3.92 b	37.76±9.75 c	66.70±3.45 a	69.01±0.44 a	67.13±3.16 a	67.80±2.00 a	68.33±2.17 a
Linolenic acid (%)	2.82±0.31 d	2.00±0.34 e	3.49±0.37 c	3.05±0.23 cd	3.89±0.51 b	4.65±0.42 a	4.75±0.53 a
TSFA	9.99±0.47 b	9.33±0.34 cd	10.01±0.54 b	9.63±0.23 bc	9.09±0.35 d	9.79±0.36 b	10.51±0.33 a
TUFA	90.01±0.47 c	90.67±0.34 ab	89.99±0.54 c	90.37±0.23 bc	90.91±0.35 a	90.22±0.36 c	89.49±0.33 d

Different small letters in a row indicate significantly different mean values at $p < 0.05$. TSFA: Total saturated fatty acid, TUFA: Total unsaturated fatty acid.

Table S4. Factor loadings and contributions of variables in the first five principal components.

Trait	PC1		PC2		PC3		PC4		PC5	
	**FL**	**%**	**FL**	**%**	**FL**	**%**	**FL**	**%**	**FL**	**%**
Days to flowering	0.59	5.47	-0.22	1.22	0.49	10.95	-0.49	15.77	-0.09	0.74
Days from flowering to maturity	-0.41	2.71	-0.18	0.87	-0.30	3.94	0.76	38.04	0.06	0.38
Days to maturity	0.35	1.98	-0.55	7.71	0.36	5.84	0.22	3.12	-0.05	0.26
Seeds per pod	-0.44	3.06	-0.19	0.89	0.17	1.25	-0.56	20.61	-0.01	0.02
Pods per plant	-0.53	4.54	-0.38	3.70	0.25	2.83	0.12	1.02	0.62	35.20
Total seeds per plant	-0.61	5.95	-0.36	3.33	0.25	2.80	-0.12	0.99	0.60	33.18
One hundred seeds weight	0.78	9.78	-0.08	0.17	0.11	0.51	0.37	9.05	-0.13	1.57
Total protein	-0.17	0.44	-0.72	13.23	0.47	9.80	0.22	3.16	-0.16	2.23
Total fat	0.62	6.15	0.23	1.36	-0.53	12.83	-0.21	2.84	0.33	9.87
Crude fiber	-0.75	9.04	-0.02	0.01	-0.18	1.41	-0.14	1.29	-0.25	5.89
Dietary fiber	-0.74	8.62	0.09	0.22	-0.34	5.14	-0.04	0.08	-0.05	0.20
Palmitic acid	-0.20	0.62	0.78	15.59	0.57	14.56	0.10	0.70	0.01	0.01
Stearic acid	0.59	5.55	0.56	8.01	-0.35	5.45	0.05	0.16	0.24	5.23
Oleic acid	0.88	12.32	-0.28	1.93	0.14	0.95	0.05	0.18	0.09	0.75
Linoleic acid	-0.86	11.91	0.22	1.26	-0.21	2.05	-0.08	0.40	-0.09	0.68
Linolenic acid	-0.86	11.85	0.13	0.44	0.25	2.73	0.12	0.99	-0.17	2.67
TSFA	-0.02	0.01	0.89	20.03	0.43	8.48	0.11	0.80	0.08	0.55
TUFA	0.02	0.01	-0.89	20.03	-0.43	8.48	-0.11	0.80	-0.08	0.55
Eigenvalue	6.27		3.92		2.21		1.51		1.10	
Variability (%)	34.82		21.78		12.27		8.38		6.09	
Cumulative (%)	34.82		56.60		68.87		77.25		83.34	

TSFA: Total saturated fatty acid, TUFA: Total unsaturated fatty acid.

Six accessions, including CP021, CP022, CP026, CP037, CP066, and CP109, outperformed in yield traits and nutritional value.
